# Identification and analysis of necroptosis-associated signatures for prognostic and immune microenvironment evaluation in hepatocellular carcinoma

**DOI:** 10.3389/fimmu.2022.973649

**Published:** 2022-08-23

**Authors:** Juan Lu, Chengbo Yu, Qiongling Bao, Xiaoqian Zhang, Jie Wang

**Affiliations:** ^1^ State Key Laboratory for Diagnosis and Treatment of Infectious Diseases, National Clinical Research Center for Infectious Diseases, The First Affiliated Hospital, Zhejiang University School of Medicine, Hangzhou, China; ^2^ National Medical Center for Infectious Diseases, The First Affiliated Hospital, Zhejiang University School of Medicine, Hangzhou, China; ^3^ Collaborative Innovation Center for Diagnosis and Treatment of Infectious Diseases, The First Affiliated Hospital, Zhejiang University School of Medicine, Hangzhou, China

**Keywords:** necroptosis, hepatocellular carcinoma, prognostic model, immunotherapy evaluation, target therapy

## Abstract

**Background:**

Hepatocellular carcinoma remains the third most common cause of cancer-related deaths worldwide. Although great achievements have been made in resection, chemical therapies and immunotherapies, the pathogenesis and mechanism of HCC initiation and progression still need further exploration. Necroptosis genes have been reported to play an important role in HCC malignant activities, thus it is of great importance to comprehensively explore necroptosis-associated genes in HCC.

**Methods:**

We chose the LIHC cohort from the TCGA, ICGC and GEO databases for this study. ConsensusClusterPlus was adopted to identify the necroptosis genes-based clusters, and LASSO cox regression was applied to construct the prognostic model based on necroptosis signatures. The GSEA and CIBERSORT algorithms were applied to evaluate the immune cell infiltration level. QPCR was also applied in this study to evaluate the expression level of genes in HCC.

**Results:**

We identified three clusters, C1, C2 and C3. Compared with C2 and C3, the C1 cluster had the shortest overall survival time and highest immune score. The C1 was samples were significantly enriched in cell cycle pathways, some tumor epithelial-mesenchymal transition related signaling pathways, among others. The DEGs between the 3 clusters showed that C1 was enriched in cell cycle, DNA replication, cellular senescence, and p53 signaling pathways. The LASSO cox regression identified KPNA2, SLC1A5 and RAMP3 as prognostic model hub genes. The high risk-score subgroup had an elevated expression level of immune checkpoint genes and a higher TIDE score, which suggested that the high risk-score subgroup had a lower efficiency of immunotherapies. We also validated that the necroptosis signatures-based risk-score model had powerful prognosis prediction ability.

**Conclusion:**

Based on necroptosis-related genes, we classified patients into 3 clusters, among which C1 had significantly shorter overall survival times. The proposed necroptosis signatures-based prognosis prediction model provides a novel approach in HCC survival prediction and clinical evaluation.

## Introduction

Primary liver cancer is ranked the sixth most commonly diagnosed cancer with almost 906,000 new cases each year. HCC is the third leading cause of cancer-related death in 2020, responsible for approximately 830,000 deaths worldwide (1). Hepatocellular carcinoma (HCC), which accounts for 75%-85% of all primary liver cancer cases, is a malignant tumor with rapid growth, high mortality and high recurrence rate after resection (2). Given the high incidence of HCC, prevention and early diagnosis in the early stage is of utmost importance (3). It is also urgent for researchers to investigate novel biomarkers that can help early diagnosis in the clinic, risk assessment, and the evaluation of therapies before and after resection.

Necroptosis is a novel type of caspase (cysteinyl aspartate specific proteinase)-independent programmed cell death that is mediated by mixed lineage kinase domain-like protein necrosomes (4), which are comprised of mixed lineage kinase domain-like protein, receptor-interacting protein kinases 1 (RIPK1) and RIPK3 (5). In this process, the tumor necrosis factor binds to its receptor (TNFR1), and RIPK1 is activated, which forms a complex with receptor-interacting serine-threonine kinase 3 (RIPK3) (6). Necroptosis is a series of pathological processes causing the swelling of organelles, cell membrane perforation membrane bleb formation and rupture, and the release of cytoplasmic contents (7), chromatin condensation and intranucleosomal DNA fragmentation. A growing body of literatures have reported that necroptosis is also involved in cancer initiation, progression, immunity, and chemoresistance (8).

Accumulating evidence has revealed that necroptosis genes play an important role in cancer initiation and progression (9, 10). Necroptosis genes are also involved in tumorigenesis, distant metastasis and immunosuppression (11). Therefore, a comprehensive understanding of the mechanism of necroptosis genes is essential to explore novel approaches for HCC diagnosis and therapies.

In this analysis, we adopted bioinformatics methods on necroptosis genes and discovered 3 hub genes linked to necroptosis associated with HCC. A prognostic prediction model was built based on necroptosis signature-related genes. Furthermore, we evaluated the clinical characteristics, the expression level of immune-related genes, and the efficiency of chemical therapies and immunotherapies between high and low risk-score subgroups. We also adjusted the necroptosis gene signature-based prognosis and survival model, which showed powerful prognosis prediction efficiency. Our model paves novel ways to establish proper treatments for HCC clinical evaluation, and offers valuable assistance with selecting properclinical treatments.

## Materials and methods

The Cancer Genome Atlas (TCGA) features genomic, epigenomic, transcriptomic, and proteomic data, providing useful information for the discovery of new tumor biological indicators and anticancer drug targets. We adopted the TCGA GDC API to fetch the RNA-seq data of liver hepatocellular carcinoma (LIHC) genomic profiles and corresponding clinical information (https://tcga-data.nci.nih.gov/tcga/), from which 343 samples were selected. We also downloaded the GSE14520 data from the gene expression omnibus (GEO) database (http://www.ncbi.nlm.nih.gov/geo), from which 212 LIHC samples were selected. In addition, we used the International Cancer Genome Consortium (ICGC)-LIRI-JP (https://www.icgc.gov/) cohort comprised of 212 LIHC samples. The dataset from TCGA was treated as the training set, and the LIHC cohort from ICGC and GSE14520 functioned as verification sets.

### Selection of necroptosis-related genes

We identified 74 necroptosis-related genes from the Molecular Signatures database (MSigDB) (http://www.gsea-msigdb.org/gsea/msigdb/index.jsp) and the reported literatures.

### Data processing

The transcription RNA-seq data from the TCGA database were processed by removing the samples that were without (1) clinical follow-up information (2), clinical survival outcome information (3), clinical pathological status information and (4) survival time of less than 30 days. The ensembles were transformed into gene symbols, and they were averaged by multiple gene symbol expression. For the GEO dataset, we downloaded the annotations from the chip platform. The corresponding gene symbol and the expression level were averaged over multiple probes mapped to the same gene symbol based on the platform information.

### ConsensusClusterPlus

In order to identify the clusters, we adopted the ConsensusClusterPlus package on necroptosis genes-based expression profiles data. The PAM algorithm (http://www-stat.stanford.edu/tibs/PAM/index.html) and 1-Spearman correlation” were used as the distance metrics. We applied 500 bootstraps as replicates, and the bootstraps contained 80% of patients in the training set. The k value was set as 2 to 10. The optimal classification was identified by calculating the consistency matrix and consistency cumulative distribution function. Finally, we determined 3 clusters based on the expression level of necroptosis genes in the TCGA-LIHC cohort.

### Construction of the least absolute shrinkage and selection operator (LASSO) Cox regression model

We identified the differently expressed genes (DEGs) between necroptosis-based clusters with false discovery rate (FDR)<0.01 and |log2FC|>1. The significant DEGs were selected (p<0.01). The LASSO cox regression analysis was adopted to narrow down the range of prognostic genes. In addition, we calculated the risk-score of each patient by risk-score=∑β_i_ x Exp _i_. All patients were classified into high and low risk-score subgroups.

### Kaplan-Meier analysis

We utilized the Kaplan-Meier analysis to evaluate the overall survival of each patient. For the Kaplan-Meier plotter analysis, all cohorts of LIHC patients were evaluated by the Kaplan-Meier plotter (http://kmplot.com/analysis/). The log‐rank tests were applied determine to significant differences among the survival curves.

### Tumor immune dysfunction and rejection (TIDE) algorithm

The TIDE algorithm (http://tide.dfci.harvard.edu/) was applied to predict the HCC cancer immunotherapy efficiency to checkpoint blockade based on the gene expression profiles (12). We evaluated the tumor associated fibroblasts (CAF), myeloid derived suppressor cells (MDSCs), and the tumor associated macrophages (TAM), which limit T cell infiltration in tumors. We also calculated the tumor immune escape indicators, such as tumor infiltration cytotoxic T lymphocyte (CTL) dysfunction, and the rejection score of CTLs by immunosuppressive factors (exclusion).

### Gene set enrichment analysis (GSEA)

We adopted the Java-based GSEA (https://www.broadlnstitute.org/gsea/) application to detect changes in the expression of target genes, which contains valuable information about the biological characteristics of genes, the relationships between gene regulatory networks, and the function and significance of genes between DEGs. The hallmark database was adopted to further analyze the enriched signaling pathways based on DEGs between different subgroups (13).

### CIBERSORT and ESTIMATE algorithm

We further employed the CIBERSORT deconvolution algorithm (https://cibersort.stanford.edu/) to qualify the abundance of specific immune cells types based on the transcriptional data of the TCGA-LIHC cohort. The Estimate-, Immune- and Stromal scores of LIHC samples were calculated by the ESTIMATE algorithm (https://bioinformatics.mdanderson.org/estimate/)

### Analysis of enriched signaling pathways

The Kyoto Encyclopedia of Genes and Genomes (KEGG) is a bioinformatics resource pool for linking genomes to biological functions. To comprehensively explore the molecular functions of DEGs, we utilized the KEGG analysis on DEGs between different clusters and recognized the most enriched signaling pathways. This analysis was performed using the R package “clusterProfiler” (https://bioconductor.org/packages/clusterProfiler/).

### QPCR analysis

Furthermore, genes were determined by real-time qPCR. The gene sequences were illustrated in supplementary table 1. LO2 and HepG2 cells lines were cultured at 37°C under 5% CO_2_ in Dulbecco’s modified eagle medium with 10% fetal bovine serum, and penicillin-streptomycin solution (1X). The total RNA was extracted from human liver cancer cell lines HepG2 and LO2 by the RNeasy mini kit (QIAGEN, Germany). Total RNA was further reverse transcribed to cDNA using the HiScript III 1st Strand cDNA Synthesis Kit (Vazyme, China) according to the manufacturer’s instructions. We also utilized the ChamQ Universal SYBR qPCR Master Mix kit (Vazyme, China) and applied biosystems (Quant Studio TMDx). Next, qPCR was utilized by a fluorescence qPCR instrument (ABI, Quant Studio TMDx, Thermo Fisher Scientific, Inc.). The target gene primers were applied in fluorescence quantitative qPCR analysis. The relative expression of target genes was calculated by fold change=2^-△△CT^.

### Statistical analysis

We applied the R 4.1.0 (https://www.r-project.org/) to present the results. We also adopted the Wilcoxon test and Kruskal–Wallis test to compare the clusters difference. In addition, we also performed the spearman for correlation analysis. In the Kaplan-Meier survival analysis, we conducted the log-rank test. For comparing the different signatures, we applied the chi-square test and Fisher’s exact test.

## Results

### Necroptosis-associated genes were classified into three subgroups with significant differences

**Figure 1:**
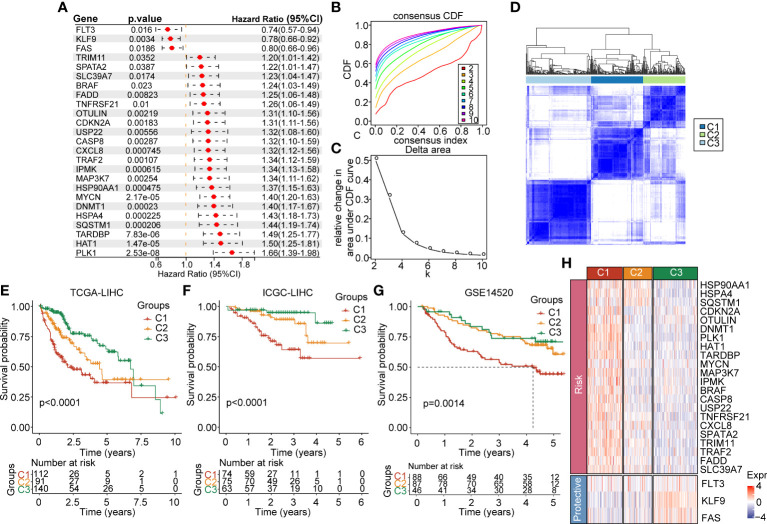
Expression landscape of necroptosis-based genes in the TCGA-LIHC cohort. **(A)** Forest map of necroptosis-related genes with prognostic significance in the TCGA-LIHC cohort. **(B)** CDF curve of the TCGA-LIHC cohort samples. **(C)** Delta area curve of consensus clustering, which indicates the relative change in area under the CDF curve for each category number k = 2 to k = 10. **(D)** Heatmap of consensus matrix k = 3 in the TCGA-LIHC cohort. **(E)** KM curve of overall survival in three clusters of the TCGA-LIHC cohort. **(F)** KM curve of overall survival in three clusters of the ICGC cohort. **(G)** KM curve of overall survival in three clusters in GSE14520 dataset. **(H)** Heatmap of expression of necroptosis genes in 3 clusters of the TCGA-LIHC cohort.

In order to link the necroptosis-associated genes with their expression, we downloaded the genomic expression information from the TCGA-LIHC dataset. To comprehensively screen out the prognosis-associated genes, we adopted the univariate cox regression analysis on necroptosis genes and identified 25 genes implicated in HCC prognosis with hazard ratio (HR) and 95% confidence interval (CI) (**Figure 1A**). Furthermore, to categorize these genes according to the prognosis signature, we adopted the consistent clustering analysis of the expression profiles of 25 prognosis-related necrosis genes included in TCGA-LIHC samples. The cumulative distribution function plots and the relative changes in area cumulative distribution function (CDF) curve results showed that k=3 could find the optimal grouping (**Figures 1B, C**). The plots (k=2 and k=4) in the supplement figures to confirm that k=3 is optimal (Supplementary **Figures 1A, B**). Furthermore, we drew the heatmaps based on the multiplied relative expression levels in 3 clusters, clusters 1 (C1), cluster 2 (C1), and cluster 3 (C3) (**Figure 1D**). We further compared the overall survival time between the 3 clusters, and figured out that C1 had the shortest survival time compared with C2 and C3 (p<0.0001) (**Figure 1E**). We also found out that C1 had the poorest survival compared with C2 and C3 (p<0.0001) in ICGC dataset and GSE14520 dataset (**Figures 1F, G**). In addition, we compared the plotted 25 prognosis-related necrosis genes in C1, C2 and C3. The results suggested that 22 risk factor genes were highly expressed in C1, which corresponded with the poorest survival in C1. Three protective genes were remarkably enriched in C3, which were linked to longer survival compared with C2 and C3 (**Figure 1H**). These results indicated that some of necroptosis genes were prognosis risk genes and others were prognosis protective genes, which might function as key regulators in HCC, aiding prognosis evaluation.

### Clinicopathological characteristics between the 3 clusters

**Figure 2:**
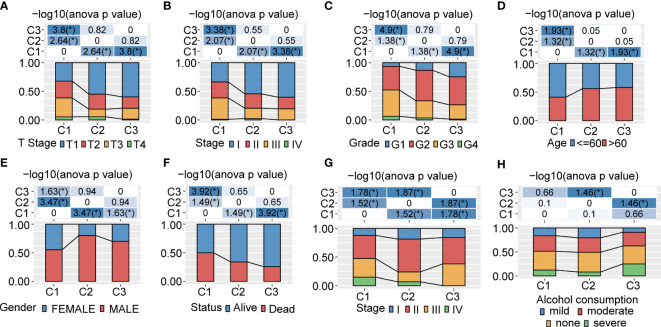
Clinicopathological characteristics between 3 clusters in the TCGA and ICGC cohorts **(A−F)**. Clinicopathological features between 3 clusters in the TCGA-LIHC cohort. The upper part of the tables represents the distribution difference between clusters. The lower part represents proportions. **(G, H)**. Clinicopathological characteristics of 3 clusters in the ICGC cohort. The upper part of the tables represents the distribution difference between clusters. The lower part represents proportions. *P<0.05.

In order to comprehensively describe the distribution of clinical and pathological features between the 3 clusters, we evaluated the clinico-pathologic staging information. For the T stage, C1 showed a significant difference compared with C3 (p<0.0001) and a remarkable difference compared with C2 (p<0.01) (**Figure 2A**). This phenomenon was also seen for the pathologic stage (**Figure 2B**), grade (**Figure 2C**), age (<60 years and >60 years) (**Figure 2D**), gender (**Figure 2E**), clinical status (**Figure 2F**), and clinical stage (**Figure 2G**). In addition, we found out that C2 and C3 were significantly different in alcohol consumption (**Figure 2H**). We also figured out that the 3 clusters showed no significant difference in N stage, M stage, viral etiology, fibrosis, viral etiology, and fibrosis (**Supplementary Figures 2A−F**). We further evaluated the clinical signatures between the 3 clusters for the ICGA dataset. The results indicated that the status in C3 was significantly different compared with C1 (p<0.01) (**Supplementary Figure 2G**). There was no significant difference between the 3 clusters in smoking, age or gender (**Supplementary Figures 2H−J**).

### Association between subtypes and mutational signatures

**Figure 3:**
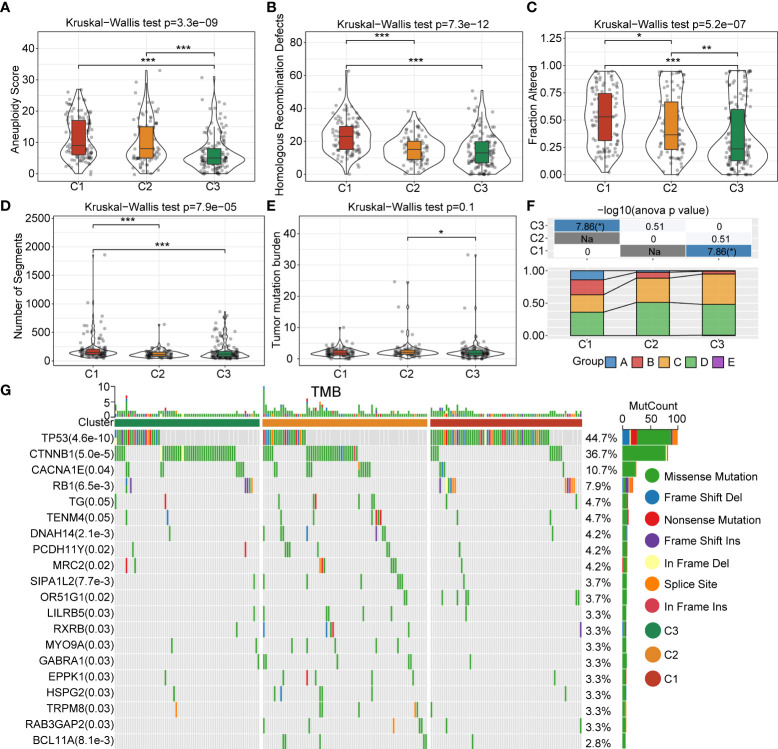
Landscapes and genomic mutation features between C1, C2 and C3 in the TCGA-LIHC cohort. **(A)** Aneuploidy score difference between 3 clusters. **(B)** Difference in homologous recombination defects between 3 clusters. **(C)** Fraction-altered percentage difference between 3 clusters. **(D)** Differences in the number of segments between 3 clusters. **(E)** Difference in the tumor mutational burden in 3 clusters. **(F)** Comparison of the proportions of three molecular subtypes and immune signature-based subtypes A–E clusters in the 3 clusters. **(G)** Somatic mutations in the three molecular subtypes (Chi-square test). *P<0.05; **P<0.01; ***P<0.001.

In order to better explore the genomic profiles between the 3 clusters, we adopted the tumor molecular signatures across 33 cancers from the TCGA cohort (14), and identified that C1 had a remarkably higher aneuploidy score (**Figure 3A**), homologous recombination defects (**Figure 3B**), fraction altered (**Figure 3C**), and number of segments (**Figure 3D**), as compared with C3. The tumor mutation burden was higher in C3 than C2 (**Figure 3E**). In addition, we classified patients into five clusters according to the immune signatures, named as A, B, C, D, and E clusters. We also compared the proportion difference of the 5 clusters, which was significantly different in C3 compared with C1 (**Figure 3F**). To gain further insights into the correlation between tumor mutations and tumor subgroups, we evaluated the clusters for mutations and found abundant somatic mutations, such as TP53, CTNNB1, CACNA1E, and RB1. The TP53 obtained higher mutation frequency in C1 (**Figure 3G**).

### Immune signatures and enriched signaling pathways between the 3 clusters

**Figure 4:**
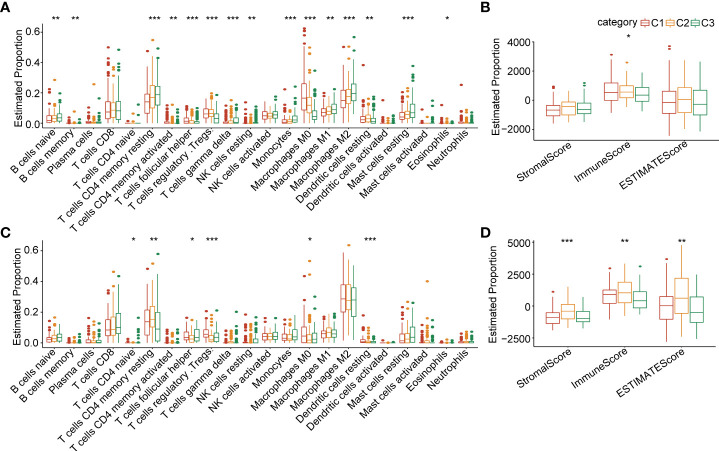
Comparisons of immune cell infiltration level in 3 clusters **(A)** The infiltration levels of 22 immune cell types between 3 clusters in the TCGA-LIHC cohort. **(B)** The ESTIMATE proportion of stromal score, immune score, and ESTIMATE score between 3 clusters in the TCGA-LIHC cohort. **(C)** The infiltration level of 22 immune cell types between 3 clusters in the ICGC cohort. **(D)** The ESTIMATE proportion of stromal score, immune score, and ESTIMATE score between 3 clusters in the ICGC cohort. *P<0.05; **P<0.01; ***P<0.001.

In order to clarify the differences in the immune microenvironment between the 3 clusters, we assessed the gene expression of immune cells to evaluate their infiltration level. The CIBERSORT tool was employed to calculate the relative abundance of immune cells in the 3 clusters. The results demonstrated that naive B cells, memory B cells, resting memory CD4+T cells, activated memory CD4+T cells, helper follicular T cells, Tregs (regulatory T cells), gamma delta T cells, resting NK cells, monocytes, M0 macrophages, M1 macrophages, M2 macrophages, resting mast cells, and eosinophils were significantly differently expressed in the 3 clusters in the TCGA-LIHC cohort (**Figure 4A**). The estimated proportion of immune score was significantly different between C1, C2 and C3 in the TCGA-LIHC cohort, and C1 had higher immune score (**Figure 4B**). A similar situation occurred in the ICGC-LIHC cohort (**Figures 4C, D**). We adopted the GSEA analysis based on the 3 clusters. We selected candidate genes from the “Hallmark” database and settled the FDR<0.05 threshold as significantly enriched. The C1 vs C3 were enriched in 21 signaling pathways, such as myc targets, E2F targets, G2M checkpoint, and others in the TCGA database (**Supplementary Figure 3A**). There were also 25 signaling pathways that were remarkably enriched in C1 compared with C3 (**Supplementary Figures 3B, C**). We further investigated the difference in signaling pathways between the 3 clusters. The results showed that C1 was almost enriched in cell cycle activation signaling pathway, thus, we inferred that the necroptosis genes in C1 were significantly activated in cell cycle signaling pathways and tumor microenvironment regulation **(Supplementary Figure 3D**).

### Identification of differently expressed genes (DEGs) between the 3 clusters and necroptosis-related hub genes

**Figure 5:**
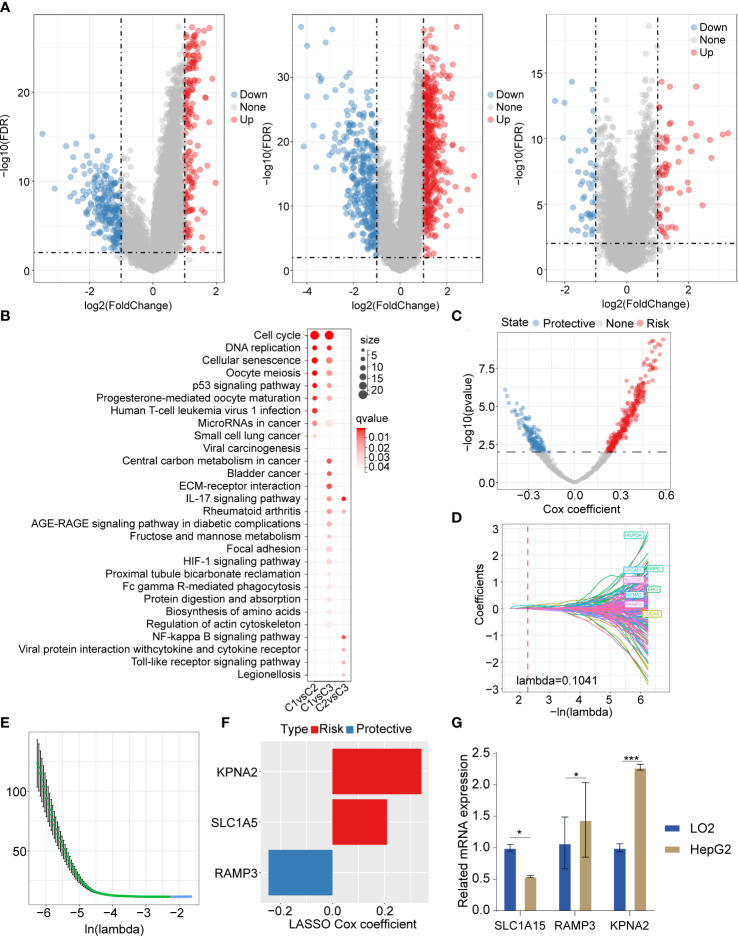
The DEGs and their enriched signaling pathways between 3 clusters, and DEG-based prognostic model construction **(A)** Volcano plot of DEGs between C1 vs C2, C1 vs C3, and C2 vs C3. **(B)** The upregulated DEGs based enriched signaling pathways in 3 clusters. **(C)** The volcano plot showed promising candidates among DEGs between 3 clusters. **(D)** The trajectory of each independent variable with the change of lambda. **(E)** The confidence interval varying under lambda. **(F)** The LASSO cox regression identified 3 necroptosis-related gene signatures. **(G)** The qPCR results showed that the 3 necroptosis-related genes were differently expressed in LO2 and HepG2. The LO2 cell lines and HepG2 cell line were obtained from ATCC (https://www.atcc.org/). *P<0.05; ***P<0.001.

In order to investigate the DEGs between the 3 clusters, we employed the “limma” package (FDR<0.01 and |log2FC|>1). We identified 159 upregulated DEGs and 209 downregulated genes between C1 and C2. We also recognized 431 upregulated DEGs and 339 downregulated DEGs between C1 and C3. There were 65 upregulated genes and 46 downregulated genes between C1 and C2 (**Figure 5A**). Furthermore, we applied the R package “clusterprofiler” for the genes selected in the previous step. The KEGG analysis showed that the upregulated DEGs between C1 and C2 were abundantly enriched in cell cycle, DNA replication, cellular senescence, P53 signaling pathway, and so on. The upregulated DEGs between C1 and C3 were mainly enriched in cell cycle signaling pathways and metabolic signaling pathways. The upregulated DEGs between C2 and C3 were remarkably enriched in inflammation-associated signaling pathways such as IL-17 signaling pathways, NF-kappa B signaling pathway, viral protein interaction with cytokine and cytokine receptor, Toll-like receptor signaling pathway, etc. (**Figure 5B**). To identify the significant necroptosis DEGs between the 3 clusters, we used the “limma” package and ultimately identified 836 genes. Furthermore, we applied univariate regression analysis and recognized 426 prognosis-related genes, which included 285 risk factor genes and 141 protective genes (**Figure 5C**). The LASSO regression analysis was adopted to process these prognosis-related genes. The trajectory changes of these independent variable coefficients were shown in **Figure 5D**. We obtained 8 genes that could reach the optimal model (Lambda=0.1041). Then, we performed multivariate analysis using stepwise logistic regression analysis. Finally, we identified three prognosis-related necroptosis hub genes: KPNA2, SLC1A5, and RAMP3 (**Figures 5E, F**). We subsequently evaluated their mRNA expression level by qPCR. The results showed that these genes were highly expressed in the HepG2 cell line compared with LO2 (**Figure 5G**).

### Necroptosis-related prognosis risk score evaluation, model construction and validation

**Figure 6:**
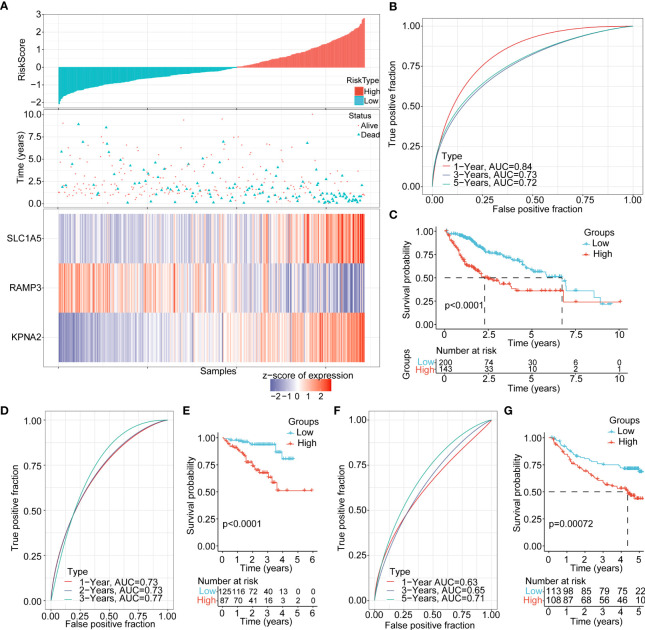
Clinical features and prognosis evaluation between the high and low risk-score subgroups **(A)** The risk score, living status, and expression landscape of 3 genes (SLC1A5, RAMP3, and KPNA2) in the TCGA-LIHC cohort. **(B)** Prognosis prediction model based on the 1-year, 3-year and 5-year AUC curves of these 3 genes (SLC1A5, RAMP3, and KPNA2). **(C)** Overall survival curves between the high and low risk-score subgroups in the TCGA-LIHC cohort **(D−G)**. AUC curves and KM curve of different risk scores between different clinicopathological subgroups in ICGC, GSE14520.

According to the risk assessment score formula, we calculated the cellular senescence-related signature score of each sample and normalized them. The risk scores of all samples in the TCHA-LIHC cohort were listed in **Figure 6A**. The ROC analysis of risk-score-based classification showed that the necroptosis prognostic signature had good predictive capability and efficiency for the one-year (AUC=0.84), three-year (AUC=0.73), and five-year (AUC=0.72) periods (**Figure 6B**). The high and low risk-score subgroups exhibited significantly different prognosis; the high risk-score group had poor prognosis compared with the low risk-score group (p<0.0001) (**Figure 6C**). To verify the stability of this model, we performed the risk score model on the ICGC and GSE14520 cohorts, and the ROC curves and overall survival curves were illustrated in **Figures 6D−G**. We found out that the higher risk-score subgroup had shorter overall survival time compared with the lower risk-score subgroup.

### Evaluation of clinicopathological features between the high and low risk-score subgroups

**Figure 7:**
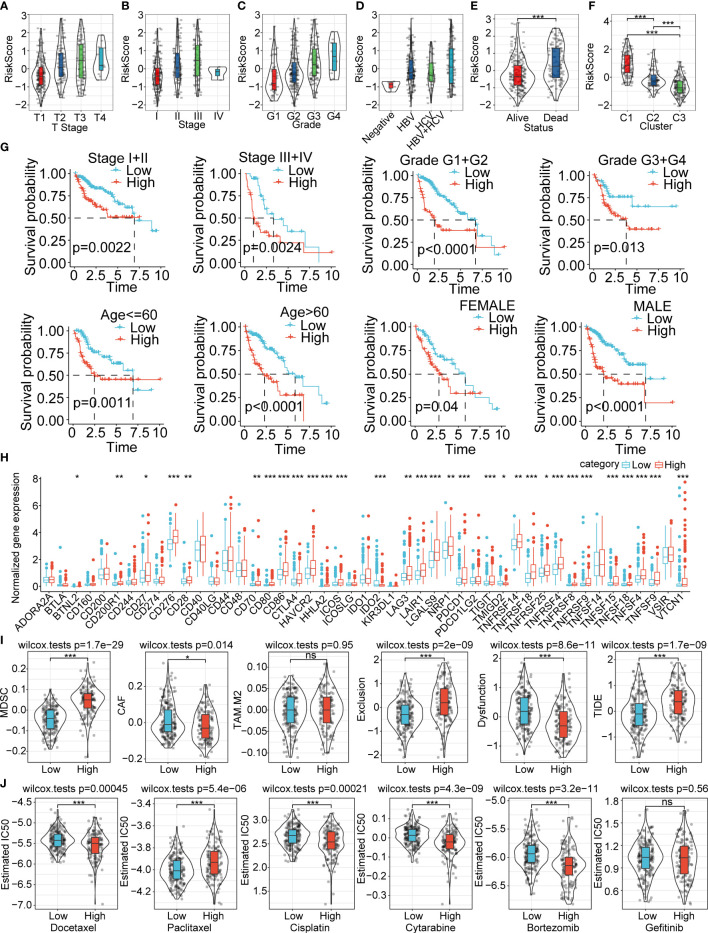
Clinical features, prognosis prediction, expression of immune checkpoint genes, immune infiltration score evaluation, and therapeutic response between the high and low risk-score subgroups **(A−F)**. Risk score difference between the various clinical feature backgrounds **(G)**. Overall survival prognosis difference between the high and low risk-score subgroups under different clinical backgrounds **(H)**. The immunological checkpoints genes were differentially expressed between the high and low risk-score subgroups in the TCGA-LIHC cohort **(I)**. Difference in TIDE score evaluation between the high and low risk-score subgroups in the TCGA-LIHC cohort **(J)**. Box plots of the estimated IC50 for docetaxel, paclitaxel, cisplatin, cytarabine, bortezomib, and gefitinib between the high and low risk-score subgroups in TCGA-LIHC. *P<0.05; **P<0.01; ***P<0.001; ns, not significant.

The risk score was significantly different for T stage (p=2e^-7^), stage (p=6.9e^-7^), grade (1.7e^-8^), viral etiology (p=0.0087), status (p=1.3e^-7^), and cluster (2.8e^-36^) (**Figures 7A−F**). There was no significant difference in N stage, M stage, fibrosis, age, and gender (**Supplementary Figures 4A−E**). Next, we explored the overall survival time between the high and low risk-score subgroups for different backgrounds of clinicopathological characteristics. The results showed that the prognosis of the high risk-score subgroup is worse than the low risk-score subgroup for stage I/II, stage III/IV, grade I/II, grade III/IV, age less than 60 years, age more than 60 years, and male gender. There was no remarkable difference between the 2 subgroups in female gender (**Figure 7G**).

### Exploration of differences in immunotherapies and chemical therapies between the two subgroups

We further compared the differences in therapies and the expression of immune checkpoint genes in the TCGA-LIHC cohort. We found out that the immune checkpoint genes were significantly highly expressed in the high risk-score subgroup compared with the low risk-score subgroup (**Figure 7H**). The high risk-score subgroup had higher MDSC, exclusion and TIDE score, which indicated higher tumor immune escape probability and lower benefits of immune therapies. The CAF and Dysfunction were much higher in the low risk-score group, and the TAM.M2 showed no difference between the two subgroups (**Figure 7I**). We also investigated the therapy response between the high and low risk-score subgroups, and the results showed that the high risk-score subgroup had higher therapy response rates and therapies sensitive to docetaxel, cisplatin, cytarabine, and bortezomib (**Figure 7J**). The estimated IC50 of paclitaxel was significantly higher in the high risk-score subgroup compared with the low risk-score subgroup (**Figure 7J**). We also explored the relative proportion of 22 types of immune cells between the 2 subgroups, and the results showed that most of the immune cells were significantly differently expressed in the high risk-score subgroup compared to the low risk-score subgroup (**Supplementary Figure 5A**). The stromal score and ESTIMATE score had higher estimated proportion in the high risk-score subgroup than the low risk-score group (**Supplementary Figure 5B**). The risk-score subgroups were significantly associated with the immune cell infiltration numbers (**Supplementary Figure 5C**). The KEGG analysis between the high and low risk-score subgroups revealed that these enriched signaling pathways were positively associated with cell cycle-related pathways, and they were negatively correlated with metabolic-associated pathways (**Supplementary Figure 5D**). In addition, we also evaluated the correlation ships between risk score and different drugs estimated IC50, the results demonstrated that the IC50 of docetaxel, cisplatin, cytarabine, and bortezomib were significantly negatively correlated with riskscore. The IC50 of Paclitaxel was positively correlated with riskscore. However, there was no significant difference between risk score and Getitinib IC50 (**Supplementary Figure 6**).

### Improvement of prognostic model based on risk score

**Figure 8:**
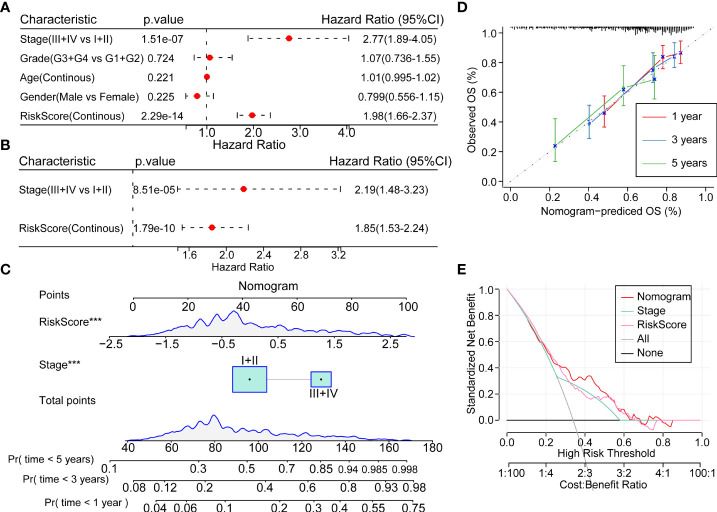
Prognostic model modification based on clinical features **(A, B)**. Univariate and multivariate cox analysis of risk-score and clinicopathological features **(C)** Nomogram showing the patient risk assessment and probability of survival in combination with the risk score and clinical characteristics. **(D)** Calibration curves of the 1, 3 and 5 years of the line graph. **(E)** Decision curve of the line graph. ***P<0.001.

In order to quantify the risk assessment and survival probability of HCC patients, we combined the risk score and other clinicopathological features to establish a nomogram. The results showed that the risk score showed the strongest impact on survival rate prediction (**Figures 8A−C**). The calibration curve estimation model demonstrated that the predictive capacity of this model for the one-year, three-year and five-year periods coincided with the standard curve. These results indicated that this nomogram possessed powerful survival prediction ability (**Figure 8D**). We also adopted the DCA to estimate the efficiency of this model, and the results suggested that the risk score and nomogram had a strong ability to predict overall mortality (**Figure 8E**).

## Discussion

The initiation and development of HCC is a multistep process, which includes early virus infection, fibrosis and hepatic cirrhosis, and eventually HCC (15). Necroptosis has been reported as a crucial event in cell death that is under pathophysiological regulation (16). It constitutes a cascade of reactions, which is triggered by the activation of receptor interacting protein kinase 3 (RIPK3) and a series of mixed lineage kinase-like domains (MLKL), which play vital roles in injury repair, pathological remodeling, chronic inflammation response, and cancer progression (16). This process, which is a form of inflammation-related programmed cell death, may have evolved into the innate immune mechanism that complements apoptosis to eliminate pathogens (17, 18). Studies have uncovered that necroptosis might also be involved in the innate immune mechanism that achieves cell death to eliminate pathogens (19). The transformation of apoptosis into necroptosis has the potential to lead to apoptosis resistance. Growing studies have reported that necroptosis combined with immune checkpoint inhibitors (ICIs) remarkably augmented antitumor capability, even in ICI-resistant tumors (20), where they play important roles in the tumor microenvironment. Sirtuin 3 functions as an important mitochondrial stress-reactive protein, which facilitates necroptosis through the deacetylation process of mutant TP53 in small cell lung cancer (21). Necroptosis-related core proteins are involved in multiple functions, directly connecting necroptosis and cell cycles (22).

Although necroptosis has been explored extensively in cancers, prognostic models based on necroptosis-related DEGs have not been fully discovered. Wu et al. also adopted the public database and bioinformation analysis methods to analysis the necroptosis genes signatures in PAAD. They found that ten PAAD prognostic markers, such as MET, AM25C, MROH9, MYEOV, FAM111B, Y6D, and PPP2R3A, were overexpressed in high-risk subgroup. They observed that CASKIN2, TLE2, USP20, SPRN, ARSG, MIR106B, and MIR98 were substantially expressed in low risk score subgroup (23). Their results were different with our results. Wang et al. also validated the necroptosis-related prognostic model in uveal melanoma (24). They classified patients into high and low clinical significance of necroptosis subgroups. The high necroptosis genes expression subgroup had a poor prognosis compared with low necroptosis genes subgroup (24). In addition, Xie et al. identified the necroptosis-related long noncoding RNAs in triple-negative breast cancer, and the subgroup with high nine necroptosis-related long noncoding RNAs signature score had poor prognosis (25). In this study, we constructed a novel prognostic model based on necroptosis-associated genes KPNA2, SLC1A5, and RAMP3. KPNA2 (26), a nuclear import factor, functions as an oncogene in cervical squamous cell carcinoma (27) and nasopharyngeal carcinoma (28, 29). In addition, SLC1A5 acts as a high-affinity transporter of L-glutamine to enhance the growth of epithelial and tumor cells in culture (30). It activates the ROS-scavenging enzyme glutathione peroxidase in cancer proliferation and migration (31). SLC1A5 (32) belongs to the Na^+^-dependent apoptosis-related specific protein family of amino acid transporters in lung cancer cells. It has roles in glutamine uptake and supporting the cell malignant capabilities of lung cancer cells’ RAMP3 function as a single transmembrane-spanning protein (33), which acts as molecular chaperone and allosteric modulator of G-protein-coupled receptors and their signaling pathways (34). Studies have reported that RAMP3 is highly expressed in a number of cancers, such as glioblastoma, renal carcinoma and breast cancer. Researchers have also highlighted that RAMP3 could mediate pramlintide-induced glycolysis inhibition and induce reactive oxygen species and apoptosis in p53 deficient thymic lymphomas (35). In the current analysis, we adopted the DEGs associated with necroptosis signatures, and constructed a KPNA2, SLC1A5 and RAMP3 model to evaluate the clinical features and prognosis in HCC, which provide key clues for acknowledging the role of necroptosis-related DEGs in HCC.

Obviously, there are some limitations of this analysis, as we adopted bioinformation methods based on a public database. It is also of great importance for researchers to deeply explore the mechanisms of necroptosis and HCC progression.

Overall, in this analysis, we deciphered that this model could not only precisely predict the survival probability of HCC but also highlight the key roles of antitumor immunity and drug sensitivity.

## Data availability statement

The original contributions presented in the study are included in the article/**Supplementary Material**. Further inquiries can be directed to the corresponding author.

## Author contributions

JL and CY designed and guided the study. JL and QB wrote and edited the manuscript. XZ and JW helped with reviewing the manuscript and collecting the references. All authors contributed to the article and approved the submitted version.

## Funding

This study was funded by the National Key Research and Development Program of China (2021YFC2301800), the National Nature Science Foundation of China (U20A20343) and the Independent Project Fund of the State Key Laboratory for Diagnosis and Treatment of Infectious Diseases, the National Key Research and Development Program of China (2022zz10).

## Conflict of interest

The authors declare that the research was conducted in the absence of any commercial or financial relationships that could be construed as a potential conflict of interest.

## Publisher’s Note

All claims expressed in this article are solely those of the authors and do not necessarily represent those of their affiliated organizations, or those of the publisher, the editors and the reviewers. Any product that may be evaluated in this article, or claim that may be made by its manufacturer, is not guaranteed or endorsed by the publisher.

## Supplementary material

Supplementary Figure 1 **(A, B)** Consistent clustering analysis showed that the plot of k=2 and k=4, samples were classified into two or four clusters.

Supplementary Figure 2
**(A−D)**. The clinical pathological features, N stage, M stage, viral etiology, and fibrosis between the 3 clusters showed no significant differences in the TCGA-LIHC cohort. **(E−J)**. Viral etiology and fibrosis condition, smoking, age, gender, and status between 3 clusters in the ICGC cohort.

Supplementary Figure 3 The enriched signaling pathways between 3 clusters **(A)**. The GSEA analysis showed that the enriched signaling pathways between C1 and C3 were cell cycle and associated signaling pathways in the TCGA-LIHC cohort **(B)**. Bar plot illustrating the enriched signaling pathways between C1 and C3 in the TCGA-LIHC and ICGC cohorts. **(C)**. GSEA analysis showing the enriched activated signaling pathways between 3 clusters in the TCGA-LIHC cohort **(D)**. GSEA analysis illustrating the enriched activated signaling pathways between 3 clusters in the ICGC cohort.

Supplementary Figure 4 Risk score difference between different clinical feature-based subgroups **(A−E)**. Risk score difference between subgroups based on N-stage, M-stage, fibrosis, age, and gender.

Supplementary Figure 5 Comparison of immune microenvironment between the high and low risk-score subgroups **(A)**.The infiltration level of 22 types of immune cells between the high and low risk-score subgroups **(B)**. The estimated immune proportion of stromal score, immune score, and estimate score between the high and low risk-score subgroups **(C)**. Correlation between 22 immune cell types and risk score **(D)**. Correlation analysis between risk score and their corresponding enriched signaling pathways.

Supplementary Figure 6 The correlation analysis between the IC50 of different drugs and risk score.
