# Supplementary material for: Identification and analysis of necroptosis-associated signatures for prognostic and immune microenvironment evaluation in hepatocellular carcinoma
Source: Front Immunol. 2022 Aug 23;13:973649. doi: 10.3389/fimmu.2022.973649 (PMC9445885; doi:10.3389/fimmu.2022.973649)
Supplement: Supplementary file 1 [file Table_1.docx]

**Supplementary Table 1. The information of primers sequences for qRT-PCR assay.**

| **Primer name** | **Sequence (5'-3')** |
| --- | --- |
| SLC1A5-F | CTGATGATGAAGTGCGTGGAGGAG |
| SLC1A5-R | GGTTGACTGCTTCGAGGATGATGG |
| RAMP3-F | CAAGTTTCCCAGTGCCCAGTGAG |
| RAMP3-R | ATGGTAGCAGTTGAAGCAGTGTGAG |
| KPNA2-F | TCTTCCTACCTTAGTTCGGCTCCTG |
| KPNA2-R | ACATTGTCCACGTAGCTTCCTTCTG |
