# Supplementary figures and images for: Identification and analysis of necroptosis-associated signatures for prognostic and immune microenvironment evaluation in hepatocellular carcinoma
Source: Front Immunol. 2022 Aug 23;13:973649. doi: 10.3389/fimmu.2022.973649 (PMC9445885; doi:10.3389/fimmu.2022.973649)

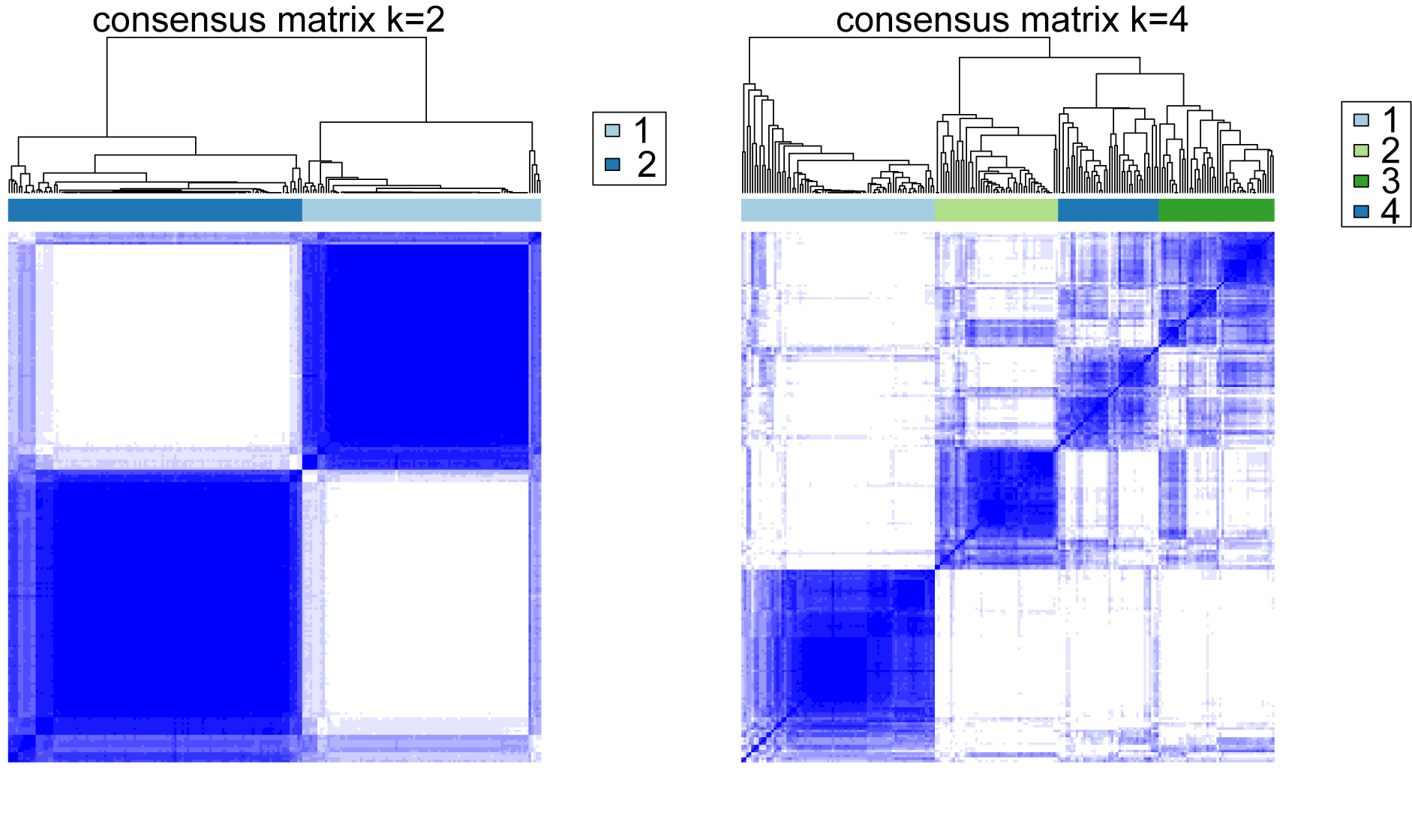

Supplement: Supplementary Figure 1 — (A, B) Consistent clustering analysis showed that the plot of k=2 and k=4, samples were classified into two or four clusters. [file Image_1.tif]

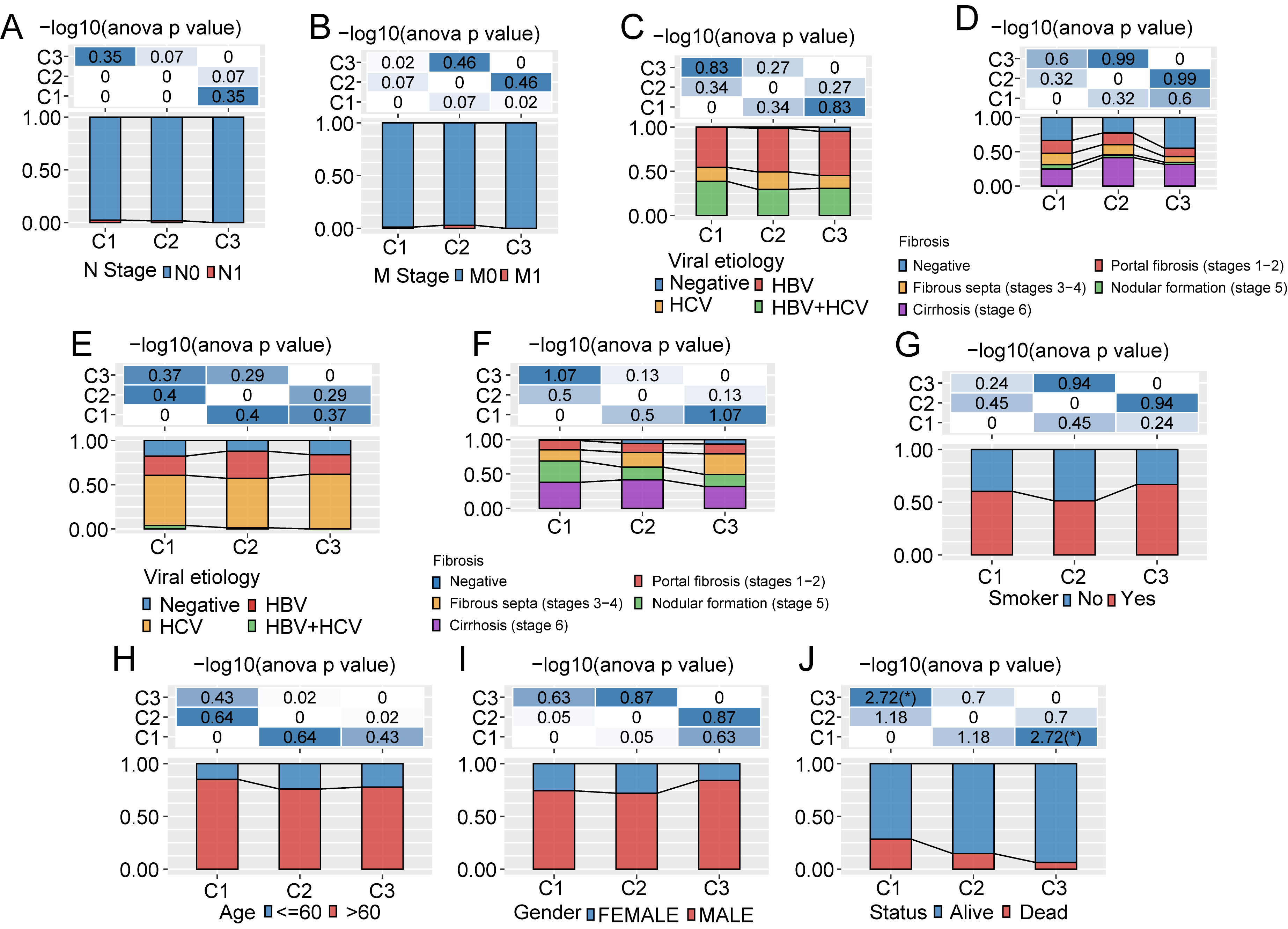

Supplement: Supplementary Figure 2 — (A−D). The clinical pathological features, N stage, M stage, viral etiology, and fibrosis between the 3 clusters showed no significant differences in the TCGA-LIHC cohort. (E−J). Viral etiology and fibrosis condition, smoking, age, gender, and status between 3 clusters in the ICGC cohort. [file Image_2.tif]

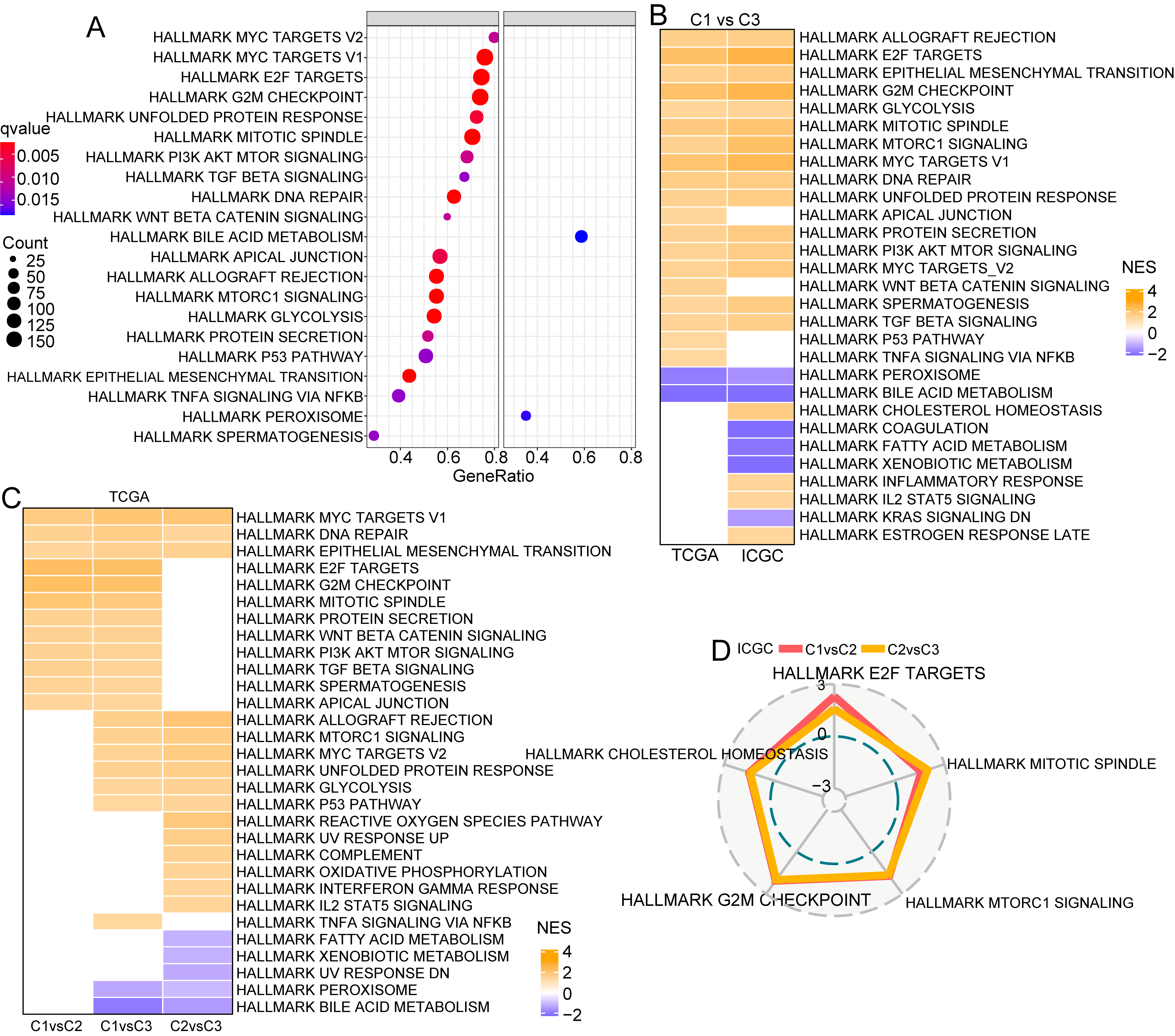

Supplement: Supplementary Figure 3 — The enriched signaling pathways between 3 clusters (A). The GSEA analysis showed that the enriched signaling pathways between C1 and C3 were cell cycle and associated signaling pathways in the TCGA-LIHC cohort (B). Bar plot illustrating the enriched signaling pathways between C1 and C3 in the TCGA-LIHC and ICGC cohorts. (C). GSEA analysis showing the enriched activated signaling pathways between 3 clusters in the TCGA-LIHC cohort (D). GSEA analysis illustrating the enriched activated signaling pathways between 3 clusters in the ICGC cohort. [file Image_3.tif]

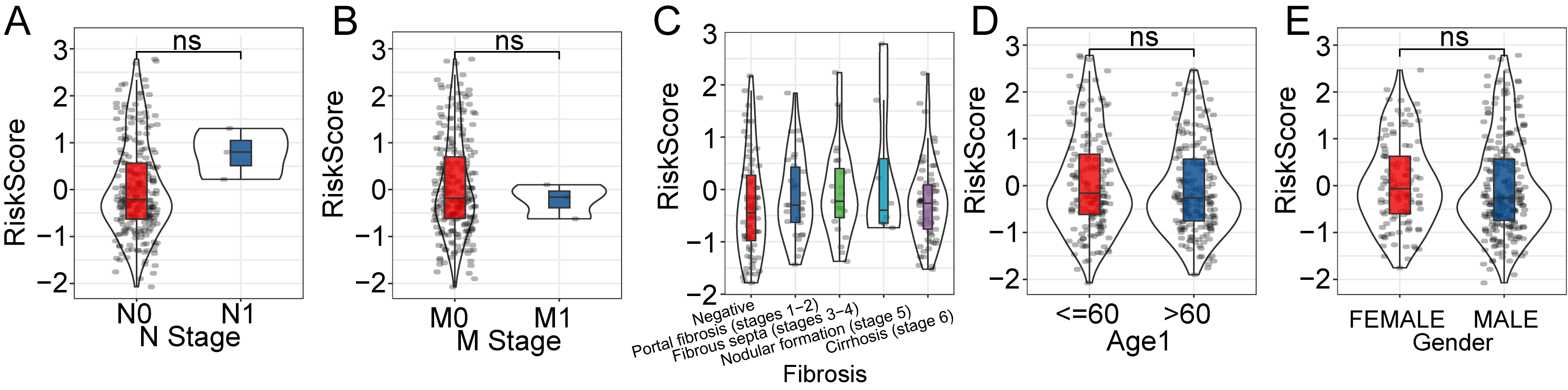

Supplement: Supplementary Figure 4 — Risk score difference between different clinical feature-based subgroups (A−E). Risk score difference between subgroups based on N-stage, M-stage, fibrosis, age, and gender. [file Image_4.tif]

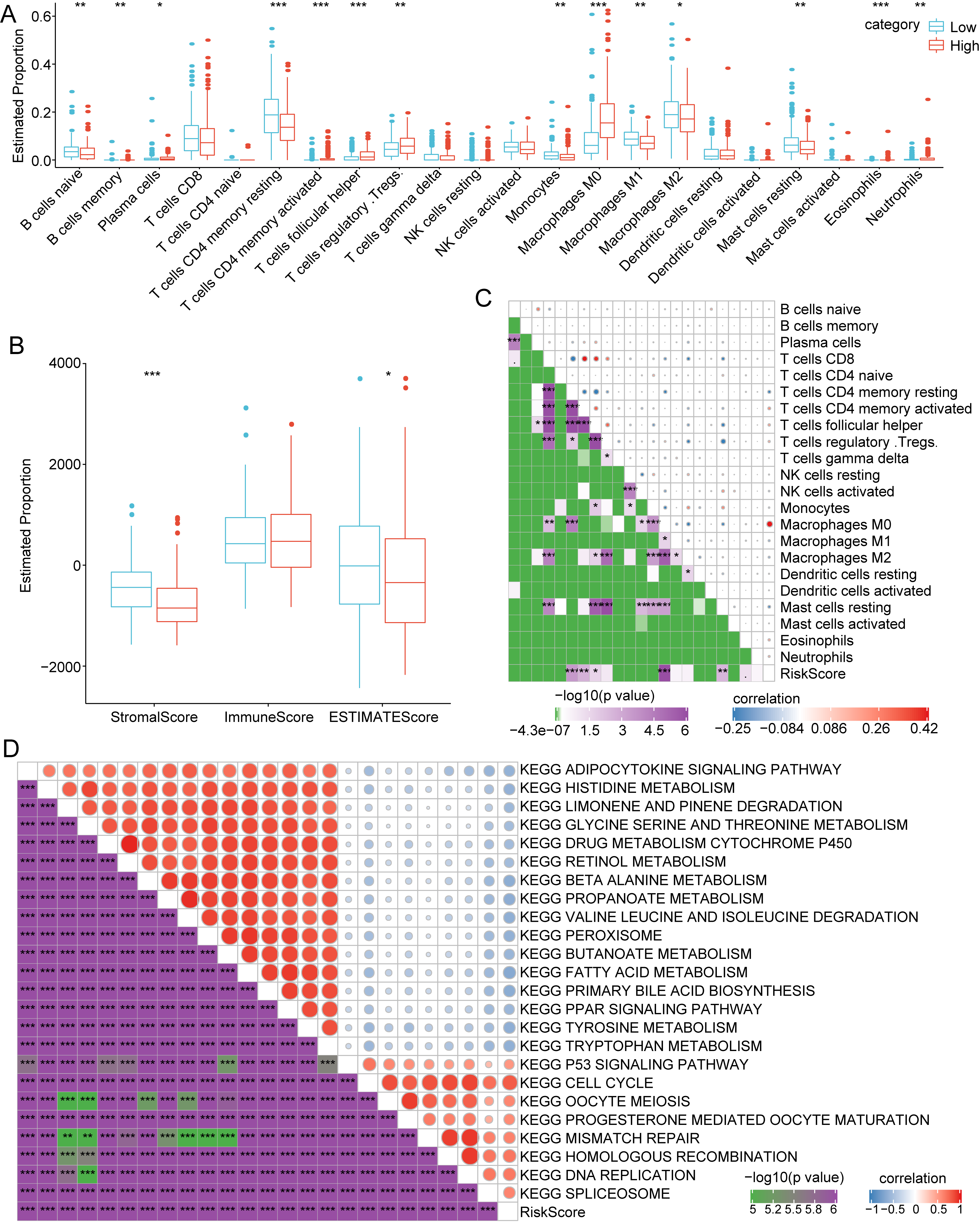

Supplement: Supplementary Figure 5 — Comparison of immune microenvironment between the high and low risk-score subgroups (A).The infiltration level of 22 types of immune cells between the high and low risk-score subgroups (B). The estimated immune proportion of stromal score, immune score, and estimate score between the high and low risk-score subgroups (C). Correlation between 22 immune cell types and risk score (D). Correlation analysis between risk score and their corresponding enriched signaling pathways. [file Image_5.tif]

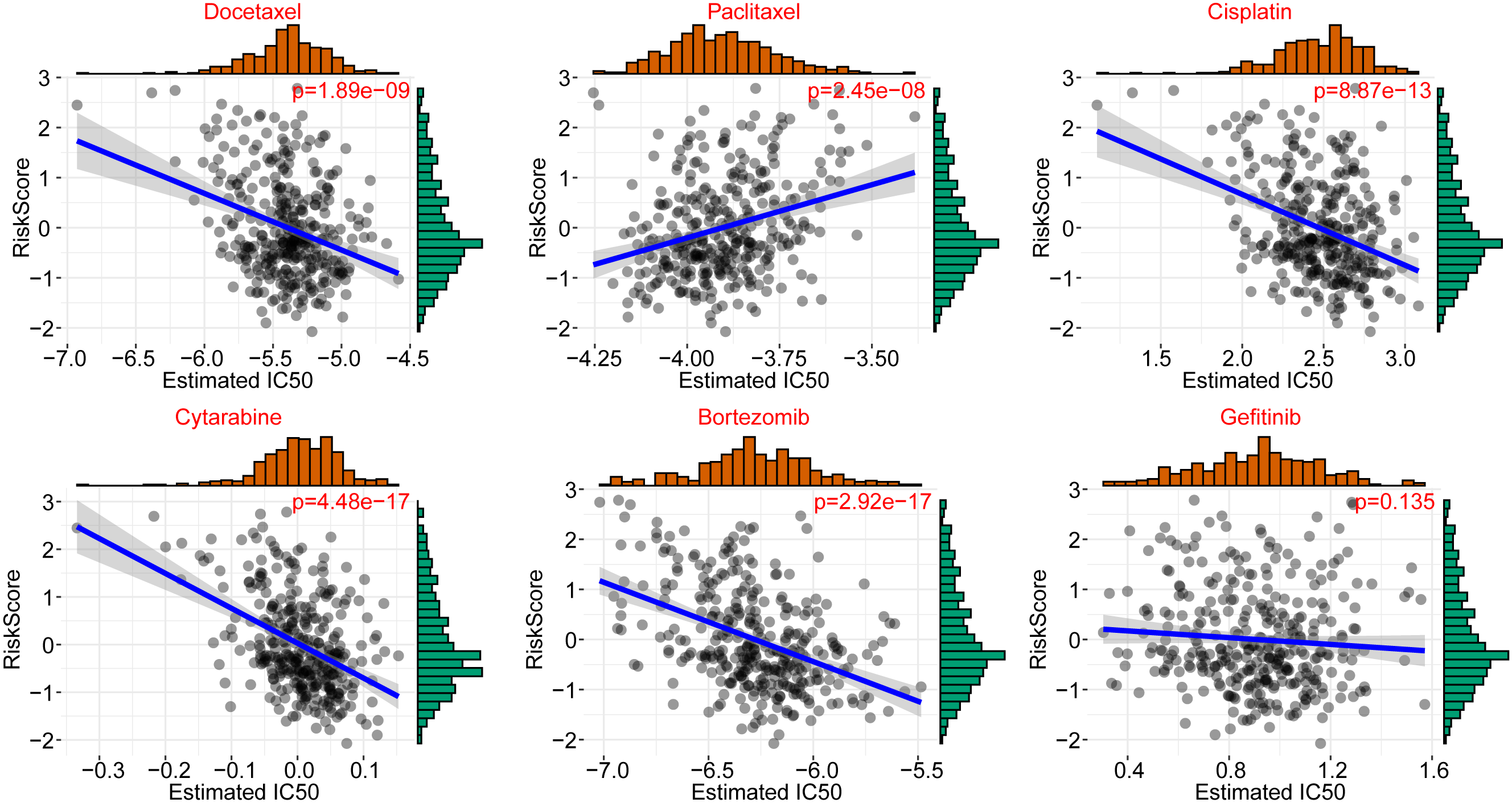

Supplement: Supplementary Figure 6 — The correlation analysis between the IC50 of different drugs and risk score. [file Image_6.tif]
